# Computing Spatiotemporal Heat Maps of Lipid Electropore Formation: A Statistical Approach

**DOI:** 10.3389/fmolb.2017.00022

**Published:** 2017-04-25

**Authors:** Willy Wriggers, Federica Castellani, Julio A. Kovacs, P. Thomas Vernier

**Affiliations:** ^1^Institute of Biomedical Engineering, Old Dominion UniversityNorfolk, VA, USA; ^2^Department of Mechanical and Aerospace Engineering, Old Dominion UniversityNorfolk, VA, USA; ^3^Frank Reidy Research Center for Bioelectrics, Old Dominion UniversityNorfolk, VA, USA

**Keywords:** molecular dynamics, trajectory analysis, multiple time scales, mutual information, distance geometry, contact network

## Abstract

We extend the multiscale spatiotemporal heat map strategies originally developed for interpreting molecular dynamics simulations of well-structured proteins to liquids such as lipid bilayers and solvents. Our analysis informs the experimental and theoretical investigation of electroporation, that is, the externally imposed breaching of the cell membrane under the influence of an electric field of sufficient magnitude. To understand the nanoscale architecture of electroporation, we transform time domain data of the coarse-grained interaction networks of lipids and solvents into spatial heat maps of the most relevant constituent molecules. The application takes advantage of our earlier graph-based activity functions by accounting for the contact-forming and -breaking activity of the lipids in the bilayer. Our novel analysis of lipid interaction networks under periodic boundary conditions shows that the disruption of the bilayer, as measured by the breaking activity, is associated with the externally imposed pore formation. Moreover, the breaking activity can be used for statistically ranking the importance of individual lipids and solvent molecules through a bridging between fast and slow degrees of freedom. The heat map approach highlighted a small number of important lipids and solvent molecules, which allowed us to efficiently search the trajectories for any functionally relevant mechanisms. Our algorithms are freely disseminated with the open-source package *TimeScapes*.

## 1. Introduction

Membrane electroporation is a biomedical technique that artificially increases the permeability of cell membranes by applying short electric pulses (Neumann et al., 1982). Electroporation by an external electric field is attributed to the opening of discrete nanometer-sized pores in cell membranes: In some plasma and biomedical experiments, pulsed fields have high power (of the order of megavolts per meter) but short duration (of the order of nanoseconds) (Kohler et al., 2015), conditions that are easily accessible to atomistic molecular dynamics (MD) simulations. Other electroporative applications such as the electroinsertion of xenoproteins or electrofusion of cells are performed in experiments at much lower voltages and over longer time scales; in these cases, statistical theories may bridge between single-event poration times derived from MD simulations and slower experimental kinetics (Böckmann et al., 2008).

**Figure 1:**
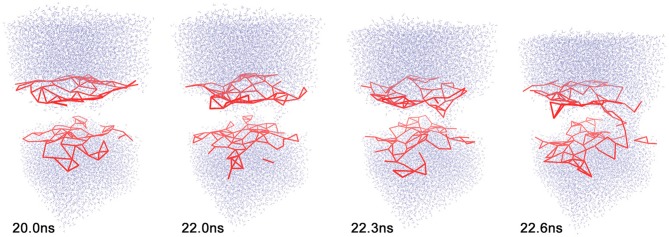
**Sequence of events during electropore formation**. Only water (top and bottom) and the coarse-grained 11 Å cutoff graph (red) of lipid phosphorus atoms are shown for clarity. Frames of trajectory 1 (see text) are shown at simulation times *t* = 20.0, 22.0, 22.3, and 22.6 ns. All molecular graphics figures in the present paper were created using the VMD program (Humphrey et al., 1996).

Direct experimental observations of electropore formation in biological membranes are not possible because of their small size and short duration. MD simulations of single-pore formation under an external electric field have consequently been of considerable interest for some time (Vernier and Ziegler, 2007; Böckmann et al., 2008; Ziegler and Vernier, 2008; Tokman et al., 2013; Kohler et al., 2015). Polar water molecules are known to play a key driving role in electroporation (Figure 1); however, no signature for pore initiation has yet been identified (Vernier and Ziegler, 2007; Ziegler and Vernier, 2008; Kohler et al., 2015), and Kohler et al. (2015) argued that a statistical framework would be needed for further development.

We have recently developed such a statistical approach for detecting allosteric signatures in protein MD simulations. *TimeScapes* is a Python-based program package that can be used to efficiently detect and characterize significant conformational changes in simulated biomolecular systems (Wriggers et al., 2009). We recently added a new functionality to TimeScapes that transforms time-domain information from MD trajectories into spatial heat maps (Kovacs and Wriggers, 2016) that can be visualized on 3D molecular structures or in the form of interaction networks. The method is multiscale in the time domain in that it uses statistical bridging between the fast, local variables recorded by MD and the slow, global rate of change of the simulated system that is characterized by a so-called activity function. In our work “activity” denotes a non-negative scalar function of time that quantifies the structural variability of the system (as introduced by Wriggers et al., 2009 and described in Kovacs and Wriggers, 2016). As simple example of an activity function is the RMS fluctuation in a sliding window. Low activity corresponds to quiescent periods of relative structural stability, whereas high activity corresponds to significant structural transitions between adjacent quiescent basins (Wriggers et al., 2009). Once the slow, global activity is quantified, the bridging between fast and slow time series can then be performed using either the Pearson cross-correlation or a nonlinear mutual information solver called Fast Information Matching (FIM).

In our recent work, we noted a potential weakness of FIM owing to the uniform Parzen window approach used in density estimation, which does not adapt well to activities that are zero-valued for some part of the simulation (Kovacs and Wriggers, 2016). In protein applications, we prefer the use of the sliding window RMS fluctuation activity that yields proper density histograms even for small systems and thereby avoids this issue. However, in the liquid (lipid or aqueous solvent) applications considered in this study, there is no stable structure that can be used as a reference for RMS fluctuation calculation. Instead, the distance geometry of intermolecular contacts is used; specifically, we use one of the two graph-based activities *TimeScapes* provides for contact networks. These graph-based activities (shown in Figure 1 and further explained below) scale quadratically with the system size and rely on a spatial coarse-graining of the structure to reduce the computational complexity, resulting in potentially zero-valued activity functions unamenable to FIM analysis. The present generalization of our heat map analysis to lipids and solvents therefore required us to develop an adaptive bandwidth allocation for the mutual information solver, which was performed separately by Kovacs et al. (2017). The resulting Balanced Adaptive Density Estimation (BADE) code for mutual information calculations is more accurate and efficient and can replace the previously used FIM code (Kovacs and Wriggers, 2016) in future versions of our *TimeScapes* package.

The “Methods” section briefly describes the theory of heat map prediction with *TimeScapes* and the adaptations that are necessary to generalize the protein-based approach to lipid and solvent dynamics. We also describe MD protocols for the electroporation simulations conducted in this study. The “Results” section first establishes activity functions that are suitable for characterizing membrane pore formation before providing examples of lipid pore formation heat maps. We explore dependencies on critical parameters of the algorithm and show heat maps of the surrounding water-ion solutions. The “Conclusions” section presents the benefits and limitations of the current framework and discusses areas for future development.

## 2. Methods

### 2.1. Transforming distance geometry time series into spatial heat maps

In this paper, we study the time-dependent distance geometry between water, ion, or lipid pairs. Let {*X*_*i,j*_(*t*)} denote pairwise distances between such “residues” (a term commonly used in MD for covalently bonded molecules that are separated by a topology or force field), where *i* and *j* are suitably chosen indices. For residues that have more than one atom, such as water molecules or lipids, pairwise distances are defined by the position of characteristic atoms (e.g., water oxygens or lipid phosphorus atoms). The time-dependent distance geometry *X*_*i,j*_(*t*) comprises “fast” variables, that is, they exhibit fluctuations on time scales of the order of the frame length of the discrete MD trajectory. Furthermore, let *a*(*t*) denote a scalar, non-negative “slow” activity function that describes the variability of the simulated system as a function of time, as described above. Finally, let *I*(*f , g*) denote a statistical measure of dependence of two discrete random variables *f* and *g* (such as Pearson cross-correlation or mutual information). The coefficient

(1) $$\begin{array}{c} R_{X,a}(i,j)=I\left(\left|\frac{dX_{i,j}(t)}{dt}\right|,a(t)\right) \end{array}$$

then provides an estimate of the spatial importance of local changes in the residue network for the global activity. In this work we are using absolute time-differentials of the fast variables for the statistical dependence analysis with the activity; this way both fast and slow timeseries correspond to a non-negative rate of change and are compatible. *R*_*X,a*_(*i, j*) values can then be used to rank all members of the family {*X*_*i,j*_(*t*)}; this, after appropriate mapping to spatial features *i* (see below), yields a heat map of the importance of fast, local variables for slow, global activities. Our transformation of time series data to spatial images can be applied to various imaging modalities *X*(*t*). However, in this study, we restrict our discussion to the abovementioned pairwise residue distances, because the distance geometry provides a suitable characterization of interactions in the absence of a global frame of reference.

### 2.2. Lipid heat map application workflow in *TimeScapes*

**Figure 2:**
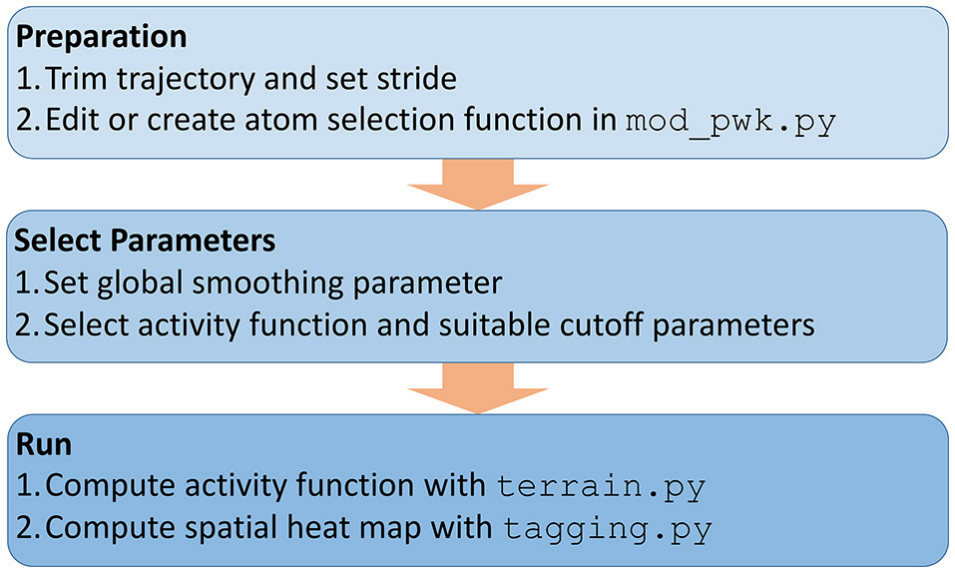
**Workflow of using ***TimeScapes*** for the lipid heat map analysis as described in the “Methods” section**.

Figure 2 shows an overview of the necessary analysis steps in our *TimeScapes* package (Wriggers et al., 2009). Before using *TimeScapes*, it is necessary to trim a trajectory to a time window of interest and to set the stride (trajectory time step). We selected time windows based on the timing of pore formation, which differed between the trajectories in this study. The end times were chosen by visual inspection when the pore size reached approximately 20% of the unit cell dimensions. The start times were chosen such that the window contained only the lead-up events immediately prior to pore formation, with full solvent perforation of the bilayer commencing at the 60% mark of the window. As a result, the poration process was normalized across the trajectory windows.

The heat maps are robust under variations in stride; however, the fastest variables captured in the analysis are limited by this choice. In our work, we selected strides of 1 and 10 ps that provided sufficient sampling of the time-dependent distance geometry.

Next, a user may have to modify the selection functions for representative atoms based on the atom and residue names defined by the force field (Figure 2). This step is necessary for the coarse-graining of the interaction networks (Figure 1). In this study, we added functions for selecting the lipid phosphorus atoms and water oxygens, which involved a straightforward edit of the available Python templates in the mod_pwk.py source file.

Few parameters must be set to run the required *TimeScapes* tools (Figure 2). An important choice is the temporal smoothing parameter that determines the temporal level of detail captured by the activity function *a*(*t*). This parameter affects both the detection of events and the bandwidth of the activity function estimation, as described in Wriggers et al. (2009). As a rule of thumb, we recommend values of approximately 5% of the trajectory window length (actual numbers are provided in the figure captions below). The “Results” section shows an evaluation of the parameter space for ensuring that the resulting lipid heat maps are robust.

**Figure 3:**
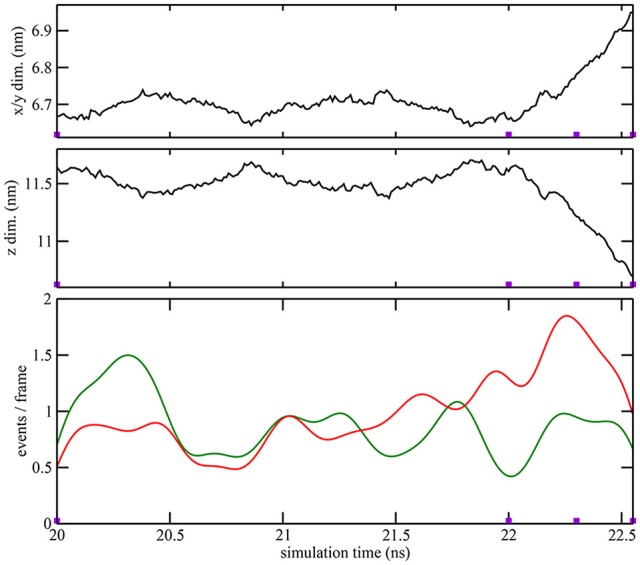
**Quantitative characterization of pore formation in ion-free trajectory 1**. The periodic unit cell dimensions (top, center) and (bottom) cutoff graph forming (green) and breaking (red) activities are shown as a function of simulation time. The graph cutoff parameters were 11 and 13 Å, and the smoothing parameter was 200 ps. The violet markers indicate the times of the four snapshots shown in Figure 1.

**Figure 4:**
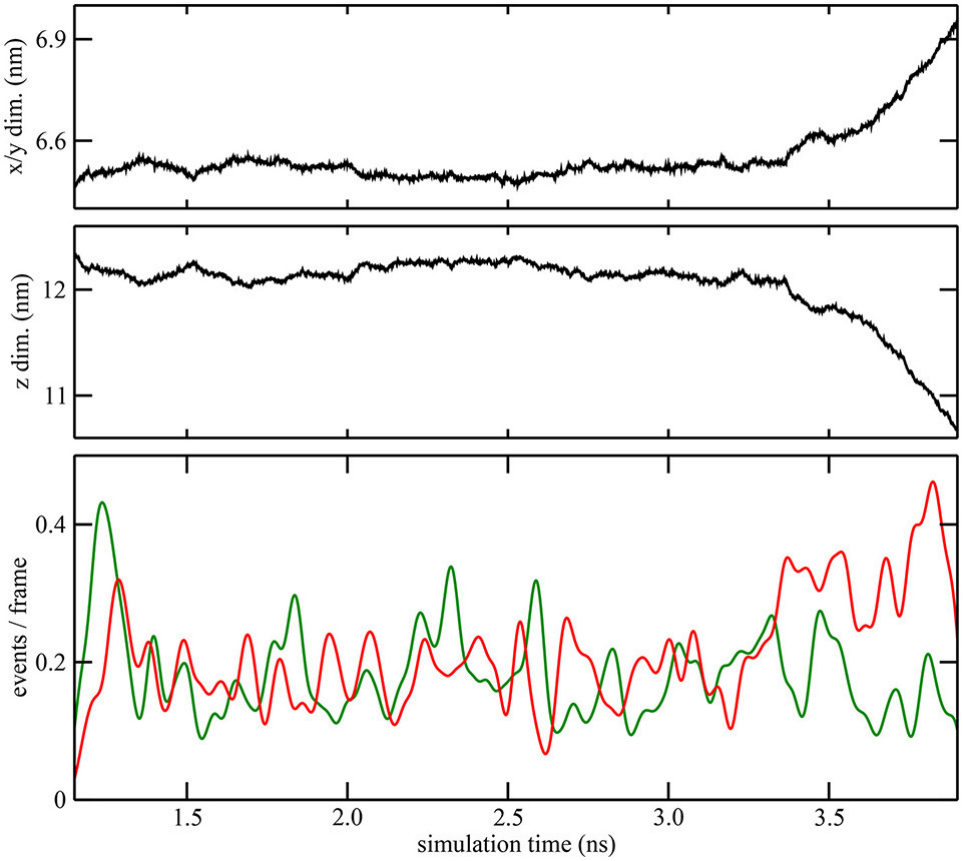
**Quantitative characterization of pore formation in ion-containing trajectory 2**. The periodic unit cell dimensions (top, center) and (bottom) cutoff graph forming (green) and breaking (red) activities are shown as a function of simulation time. The graph cutoff parameters were 11 and 13 Å, and the smoothing parameter was 50 ps.

The global activity of the system *a*(*t*), which is required for heat map analysis, can be computed from changes in a distance cutoff-based adjacency graph or from a so-called Generalized Masked Delaunay graph (Wriggers et al., 2009). In this work, we chose a cutoff graph (Figure 1) of lipid phosphorous atom distances because it decomposes structural changes into separate contact-forming and -breaking activity of adjacent lipids (Figures 3, 4). The Generalized Masked Delaunay graph (not discussed here) is less affected by distances and is thus less capable of differentiating between forming and breaking events (Wriggers et al., 2009). *TimeScapes* also supports the calculation of RMS-fluctuation-based activity of Cartesian coordinates in a Gaussian-weighed sliding window. However, this approach requires a global frame of reference for least-squares fitting, such as a protein structure, which is not available in our liquid systems. Consequently, among the three activity functions available in *TimeScapes*, we only use the cutoff graph that can be computed using the terrain.py tool (with the user-provided phosphorus selection function). This graph requires the setting of two distance cutoff values for the event detection buffer. As a rule of thumb, the cutoff values should reflect the nearest-neighbor distances in the coarse grained model (i.e., lipid phosphorus atoms; Figure 1).

Finally, after computing the activity function using terrain.py, the program tagging.py uses it to compute the positive symmetric matrix *R*_*X,a*_(*i, j*) of the ranking coefficients between the time series *X*_*i,j*_(*t*) and *a*(*t*) (Figure 2). This matrix quantifies the statistical dependence of every residue pair (*i, j*) with the activity function *a*(*t*). As discussed in Kovacs and Wriggers (2016), the matrices *R*_*X,a*_(*i, j*) show a banded structure owing to the global nature of the statistical relationship between the activity and the concomitant change in distances from a particular residue to neighboring residues (Shaw et al., 2010). The banded structure of this matrix allows us to compress the columns of *R*_*X,a*_(*i, j*) to their average *R*_*X,a*_(*i*), so we can visualize the pairwise heat maps in three dimensions. We note that unlike in the earlier heat map projection to relatively stable protein structures (Kovacs and Wriggers, 2016), the heat maps in the present paper are projected to lipid or solvent molecules that undergo diffusive motion throughout the trajectory. Because we are essentially drawing an image on a moving canvas, it is valuable to visualize the results on a time-dependent trajectory instead of static structures.

The adaptation of *TimeScapes* to lipid systems in this study also required some updating of the source code, mainly to deal with the periodic boundaries and to read the unit cell box dimensions. The updated code will be released with version 1.5 at our web site, http://timescapes.biomachina.org.

### 2.3. Molecular dynamics simulations of electroporation

Atomic-scale MD simulations of a symmetric phospholipid bilayer were performed using the GROMACS 4.6.6 software package (van der Spoel et al., 2005) on the Turing High Performance Computing cluster at Old Dominion University (Old Dominion University, 2017). A system containing only lipids and approximately 12,000 water molecules was created using the MemBuilder tool (Ghahremanpour et al., 2014). Four trajectories were generated for this paper. Trajectory 1 contained no ions. For trajectories 2–4 the built-in GROMACS function genion was used to replace bulk water molecules with Ca^2+^ and Cl^−^. This generated an ionic solution comprising 20 calcium ions, 40 chloride ions, and approximately 12,000 water molecules. The CHARMM36 force field and TIP3P water model were used. The charge and size for both calcium and chloride ions were rescaled in accordance with Kohagen et al. (2014) to improve the ion-water interactions and to avoid unrealistic ion clustering. The simulation volume for both systems contained 128 (64 per leaflet) lipid molecules—1-palmitoyl-2-oleoyl-sn-glycero-3-phosphatidylcholine (POPC)—with initial box dimensions of approximately 7 × 7 × 12 nm. The system was equilibrated for 1,500 ns to allow calcium-phospholipid binding (Vernier et al., 2009) and to stabilize the area per lipid. All simulations were performed under the NPT ensemble. A temperature of 310 K was maintained using a velocity rescaling algorithm (Bussi et al., 2007). A pressure of 1 bar was maintained using a Berendsen barostat (Berendsen et al., 1984) with relaxation time of 1 ps and compressibility of 4.5 × 10^−5^ bar^−1^ applied semi-isotropically in both normal and in-plane directions relative to the membrane. Bond lengths were constrained using the LINCS algorithm (Hess et al., 1997) for lipids and SETTLE (Miyamoto and Kollman, 1992) for water. Short-range electrostatic and Lennard-Jones interactions were cut off at 1.0 nm. Long-range electrostatics were calculated using the PME algorithm (Essmann et al., 1995), and boundary conditions were used to mitigate system size effects. The integration time step was 2 fs. An electric field of 400 MV/m was applied along the z-axis normal to the (x,y) bilayer plane. Under these conditions, pores form within approximately 3–20 ns. Trajectory 1 was run for 23.5 ns with a stride (trajectory saving time) of 10 ps. For systems containing calcium, three independent trials of lengths 5.3, 23.4, and 16.5 ns were run with a stride of 1 ps by assigning a randomized velocity to each atom after system equilibration. The trajectory windows selected to normalize the pore formation times (see above) were frames 2001–2256, 1150–3900, 19350–22100, and 13550–15300 for trajectories 1–4, respectively. The selection of these windows had the added benefit that any initial periodic box deformation of the system was already discounted (applying an electric field normal to the plane of a lipid bilayer under the NPT ensemble causes a reduction in the bilayer thickness and a corresponding change in the box dimensions, preceding and independent of pore formation).

## 3. Results

### 3.1. Activity functions relevant for pore formation

As described in the “Methods” section, one of the prerequisites of the heat map analysis is the use of an activity function that characterizes the global change of the system. This work focuses on pore formation that introduces an anisotropic pressure in the system and by virtue of the NPT ensemble yields a compression of the periodic unit cell in the z-direction and an associated elongation in the x- and y-directions. The unit cell dimensions can therefore be used as a geometric marker for pore formation, as shown in the top and center plots of Figures 3, 4 for ion-free trajectory 1 and for one of the ion systems, trajectory 2, respectively. The data for trajectories 3 and 4 were similar to those of trajectory 2 and are omitted for brevity. The four simulation times in Figure 3 can be compared visually to the corresponding snapshots in Figure 1.)

In addition to the geometric characterization of the poration process, we also computed the time-dependent cutoff graph (Figure 1) that decomposes structural changes in the lipid bilayer into separate contact-forming and -breaking events. The resulting lipid-forming and -breaking activities are plotted at the bottom of Figures 3, 4. All four trajectories showed sustained breaking activity during pore formation that is not equally compensated for by the forming of lipid contacts. Consequently, we used the lipid-breaking activity (red graphs) as a measure of pore formation in our subsequent heat map analysis. The unit cell deformation was then used as an independent measure for validation.

### 3.2. Gallery of lipid pore formation heat maps

**Figure 5:**
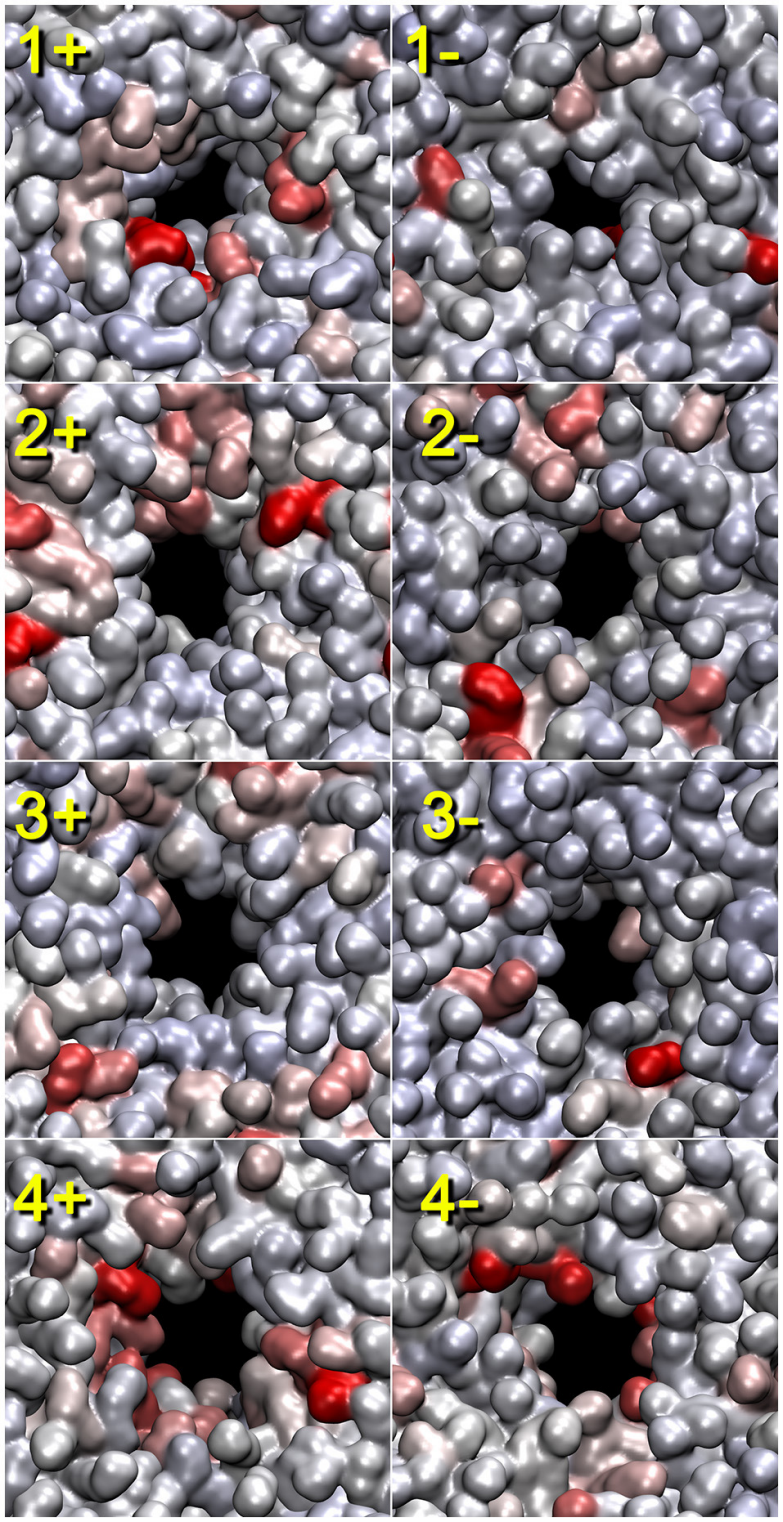
**Lipid heat maps indicating the statistical importance of individual lipids for the pore formation (as represented by the lipid contact breaking activity)**. The mutual information analysis for the lipid bilayer was conducted using tagging.py with the BADE solver described in Kovacs et al. (2017). The temporal smoothing parameter was 200 (trajectory 1) and 50 ps (trajectories 2–4). The front (+z direction) and rear (-z direction) views of the heat maps generated from the four trajectories are shown. Solvent molecules are omitted for clarity. Lipid heat maps in the present paper were rendered using QuickSurf mode in VMD (Humphrey et al., 1996) with a linear red-white-blue color scale (from high to low mutual information values). The pores and their symmetry mates were centered in the unit cell with image dimensions cropped to cell size. The 3D structures used for the rendering of the heat maps correspond to the last frame of the trajectory windows (see Section “2.3”).

The lipid heat maps shown in Figure 5 visualize the importance of individual lipids for the contact-breaking activity associated with pore formation. This figure shows mutual information heat maps of trajectories 1–4 for both sides of the bilayer (as viewed in the +z and −z directions). In many, but not all, heat maps, we observe hot spots that are clearly associated with the emerging pore (most notably in views 1+, 2+, 4+, and 4−). Some cases also show outliers that are not associated with the pore (1−, 2−, 3+, and 3−). The heat maps are very valuable because they allow a user to focus on the relatively small number of statistically significant lipids. However, a detailed inspection of the trajectories does not reveal a consistent mechanism of action of these lipids. The outliers have their head groups exposed to the solvent, and they are important for a general destabilization of the bilayer that facilitates pore formation; however, there is no indication that the outliers participate in the actual poration event. The highlighted lipids that line the pore exhibit a disruption of their contact network; however, an inspection suggests that this appears to be a passive response to the tunneling of water molecules across the membrane.

### 3.3. Validation

**Figure 6:**
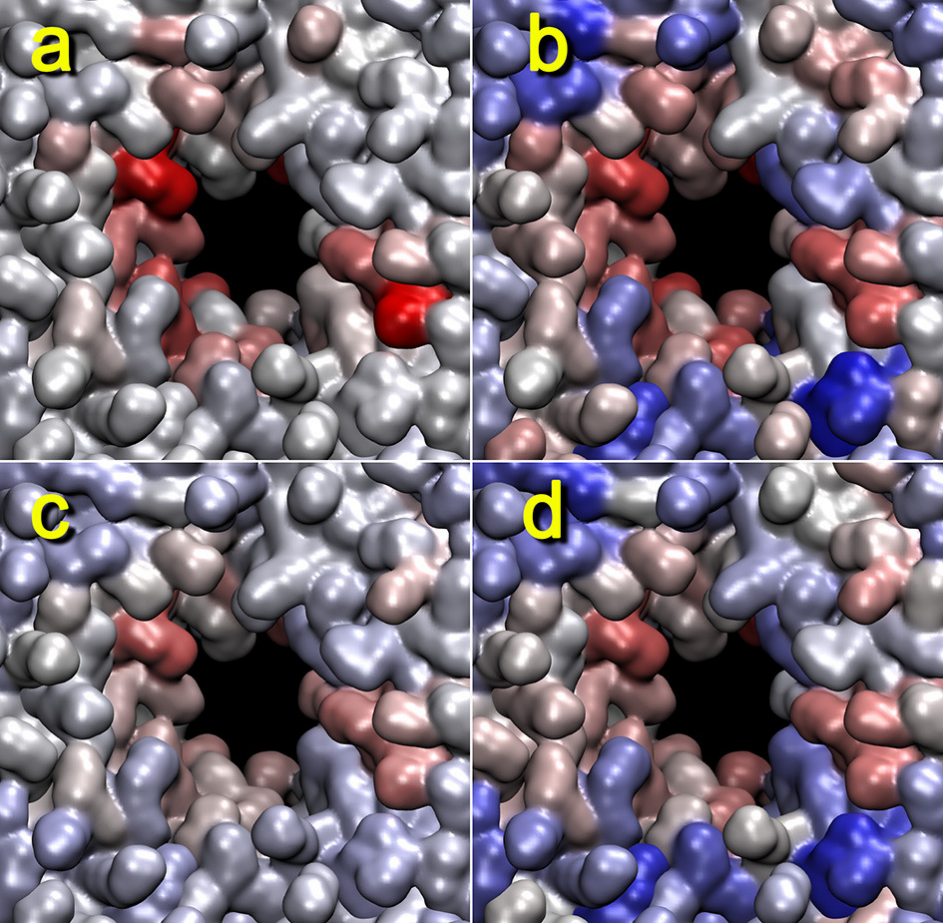
**Lipid pore formation heat map validation**. The heat maps for trajectory 4 (+z direction view) generated with mutual information **(a,c)** or Pearson cross correlation **(b,d)** against activity data from the cutoff graph **(a,b)**, z dimension of the unit cell **(c)**, or x dimension of the unit cell **(d)** are shown. The parameters are otherwise the same as those in Figure 5. When considering the box dimensions, we note that the Pearson correlation expects (positively) co-correlated features (see text). Therefore, we have used the x dimension of the unit cell (identical to the y dimension); on the other hand, in the mutual information case, this distinction did not matter, and we used the z dimension for the analysis.

We conducted a number of alternative heat map calculations to test the robustness of our approach and to validate the results shown in Figure 5. Figure 6 shows the results when replacing mutual information Figures 6a,c with the Pearson cross-correlation Figures 6b,d and when replacing the graph-based lipid activity Figures 6a,b with the box dimensions Figures 6c,d in the trajectory 4 heat map. The Pearson cross-correlation is a simple, linear measure of statistical dependence. As implemented in tagging.py, negative correlations serve to measure the noise floor and are afterwards set to zero (only positive correlations between rates of change make physical sense, so negative correlations are deemed noise). However, the mutual information captures all non-linear dependencies (including negative linear correlations). Therefore, the resulting mutual information heat maps are smoother, whereas Pearson cross-correlation heat maps show higher dynamic range. Despite these differences, the two measures show comparable features.

Remarkably, heat maps are also largely unaffected by the type of activity function used (Figure 6). As shown in Figures 3. 4, the red lipid-breaking activity graphs can be quite different from the box dimension graphs; however, the trajectory 4 heat map shows the same features in either case. This demonstrates that our contact-breaking activity is indeed a suitable measure for pore formation. Minor discrepancies in the heat maps in Figure 6 are expected because the box dimensions probe for the size of the water tunnel, whereas the breaking activities probe for lipids that weaken the bilayer.

### 3.4. Evaluation of parameter space

As discussed earlier (Figure 2), the user must select several program parameters for the analysis, and it is worthwhile to investigate how sensitive the results are to such subjective choices.

**Figure 7:**
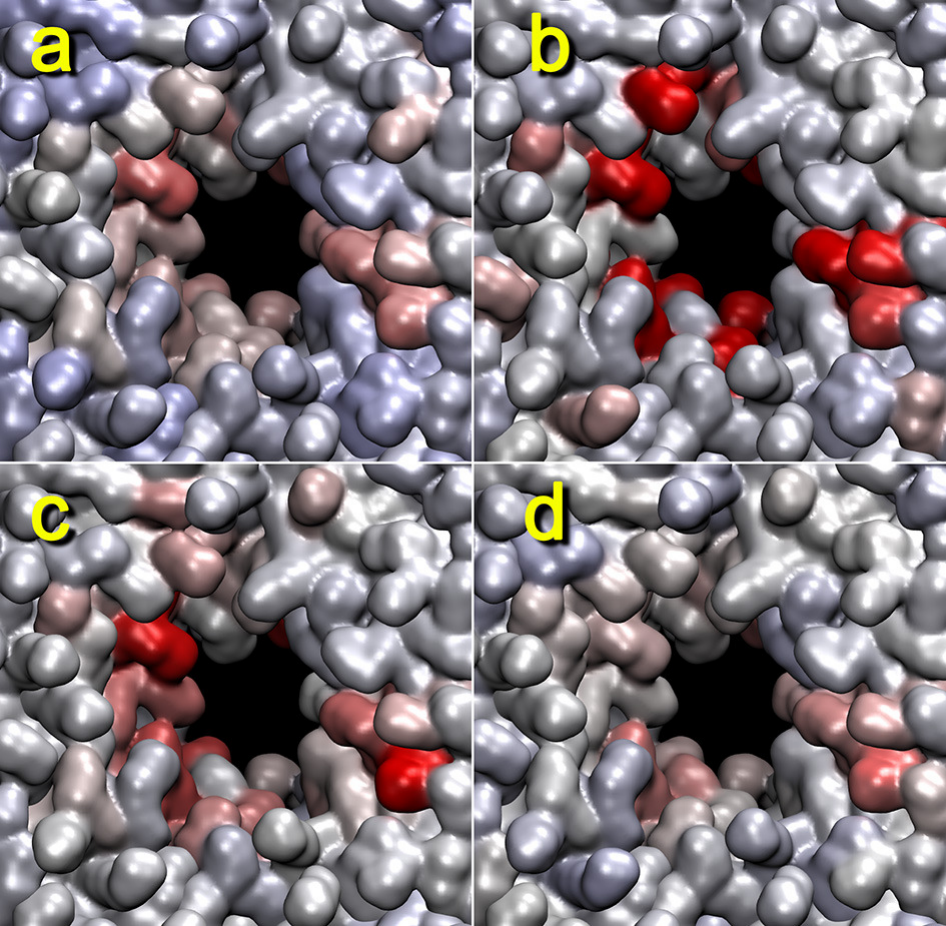
**Dependency of lipid heat maps on temporal smoothing parameter used in** terrain.py **and** tagging.py. The heat maps for trajectory 4 (+z direction view) generated with mutual information against activity data from z dimension of the unit cell **(a)**, or the cutoff graph **(b–d)** with smoothing parameters 20 **(b)**, 50 **(c)**, and 100 ps **(d)** are shown. The parameters are otherwise the same as those in Figure 5.

We have used the box dimension as a benchmark for estimating the proper contact graph smoothing parameter in Figure 7. Although the lipid-breaking activity shows slightly different results, the overall appearance should be comparable. In Figure 7, we used three smoothing parameters: 20, 50, and 100 ps. All three cases had highlighted lipids at the pore; however, the smoothing parameter of 50 ps gave the closest match with the box dimension heat map, and that of 100 ps was a close second. This is in good agreement with our earlier rule of thumb, namely, to start the analysis with a smoothing of approximately 5% of the window width.

**Figure 8:**
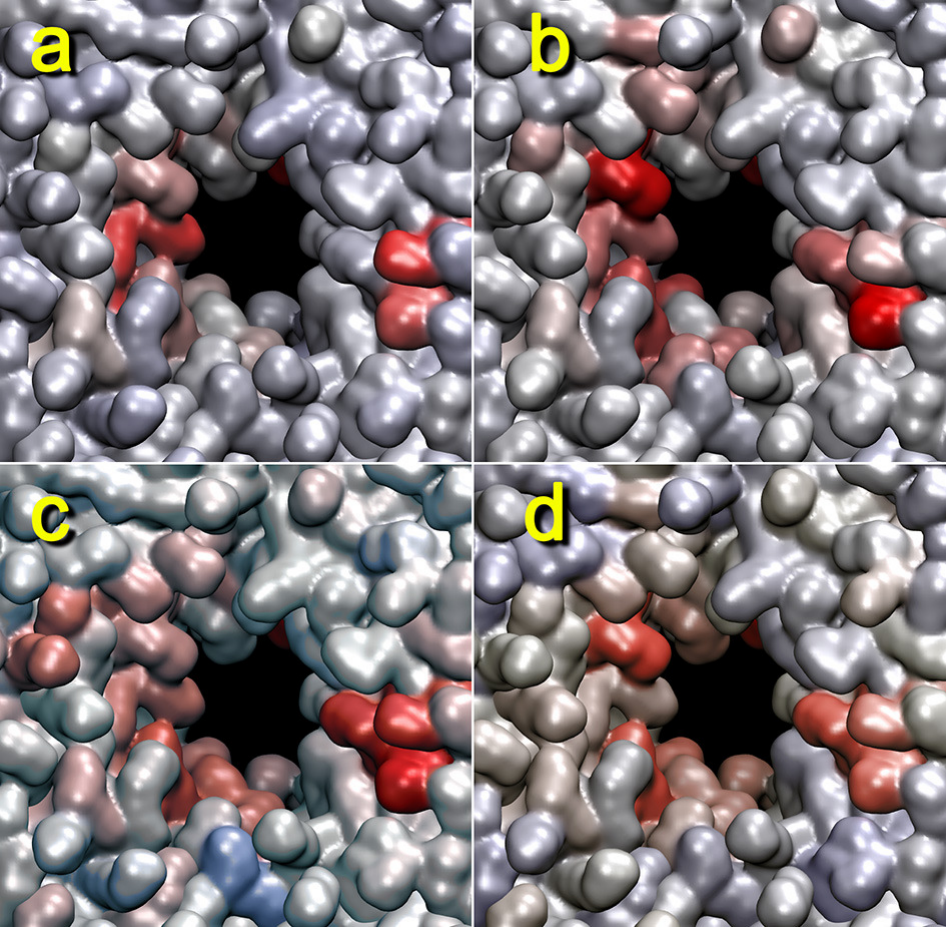
**Dependency of lipid heat maps on graph distance cutoff used in** terrain.py. The heat maps for trajectory 4 (+z direction view) generated with mutual information against the cutoff graph with buffers of 9–11 **(a)**, 11–13 **(b)**, 13–15 **(c)**, and 15–17 Å **(d)** are shown. The parameters are otherwise the same as those in Figure 2.

Figure 8 shows the dependence of the heat map on the distance cutoff values for the contact graph. As a rule of thumb, the cutoff values should reflect the nearest-neighbor distances in the coarse grained model (two values bracket a buffer zone for recrossing suppression, and the lower value is most important whereas the upper value is typically set 1–2 Å higher). For lipids, a visual inspection of the lipid phosphorus atoms suggests that a lower cutoff of 11 Å would be appropriate (Figure 1). Figure 8 shows heat maps for cutoff values of 9–15 Å (with a 2 Å buffer). The 9 Å value misses many of the lipid phosphorus contacts; however, results above 11 Å appear reasonably stable. Therefore, we used 11 Å for most of the analyses in this study.

### 3.5. Solvent pore formation heat maps

**Figure 9:**
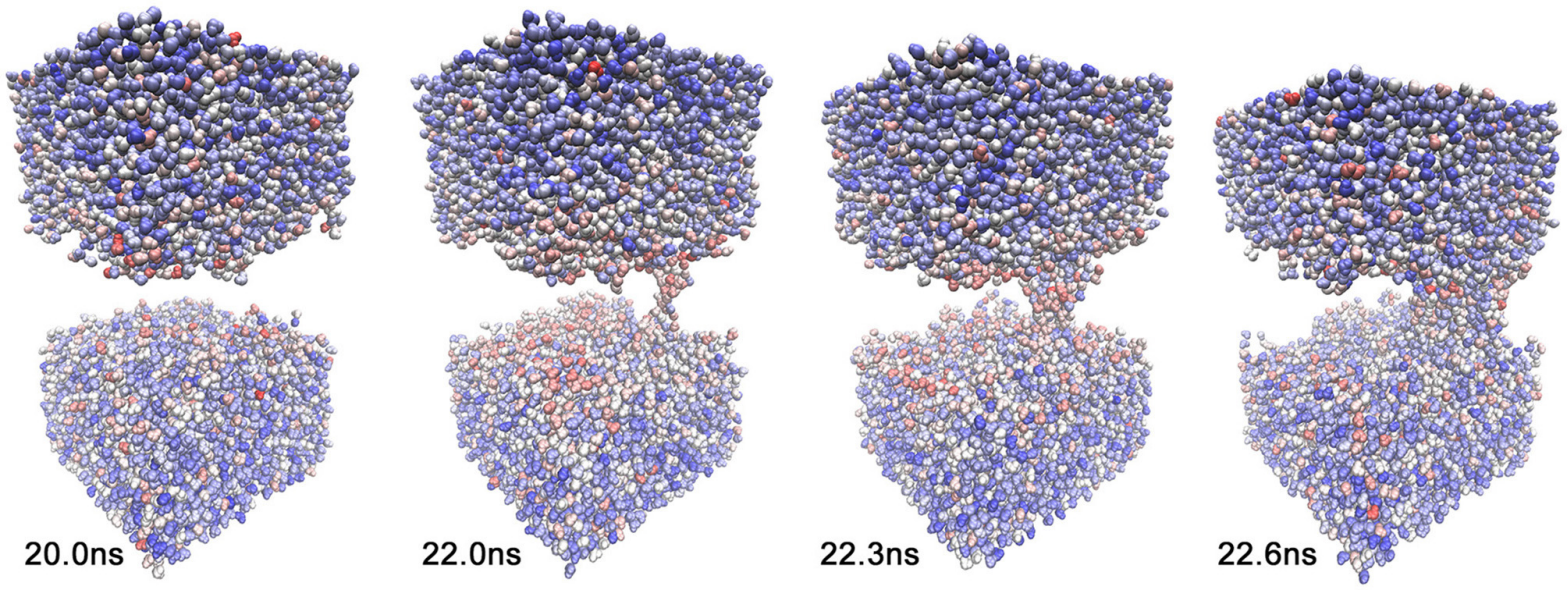
**Solvent heat map of trajectory 1 generated with mutual information against activity data from the lipid cutoff graph with smoothing parameter 200 ps**. The lipids are omitted for clarity. The snapshots were taken at the same simulation times as in Figure 1, *t* = 20.0, 22.0, 22.3, and 22.6 ns. The solvent heat map was rendered using VDW mode in VMD (Humphrey et al., 1996) with a linear red-white-blue color scale (from high to low values).

We also investigated whether we see any evidence that the global lipid dynamics, as described by the activity function, drives the solvent dynamics. Toward this end, we also generated a heat map for solvent molecules that were coarse-grained to one atom per molecule. Because we project the heat map on a moving canvas of rapidly diffusing solvent molecules, Figure 9 shows the results for trajectory 1 as a function of time. Figure 9 reveals both a temporal focusing of the solvent heat map on the pore formation time and a spatial focusing on the membrane-solvent interface (although there is no preferential association of the heat map with the emerging pore). The highlighted solvent molecules are clearly located at the lipid-solvent interface at 22.0 and 22.3 ns; however, before and after pore formation (20.0 and 22.6 ns) they are dispersed throughout the membrane by their diffusive motion. The spatio-temporal focusing shows the importance of the general weakening of interactions that precedes (or precipitates) the solvent perforation of the lipid bilayer (Figures 3, 4). Inspecting the heat map further shows that the identity and origin of the tunneling water molecules in the pore is not determined by their position before the intrusion occurs; therefore, at 22.0 and 22.3 ns there is no accumulation of heat-map highlighted waters in the pore beyond the level that is generally observed at the interface. The dispersed heat map at 20.0 ns shows that water molecules in the pore can be from the interface or from the bulk. Sometimes, interfacial water can be seen climbing past the interface region, and then, ballistic water sails in from the bulk and replaces it.

Water and ions have one disadvantage, namely, that any coarse-graining on a per-molecule basis to compute the time-dependent distance geometry *X*_*i,j*_(*t*) is rather limited. For approximately 12,000 solvent molecules, we were able to achieve manageable analysis times (of the order of days) by using a 15 Å distance cutoff for significant interactions (as an argument passed to tagging.py) to reduce the number of pairwise water interactions.

## 4. Conclusions

Over the last several years, we have developed a statistical strategy for transforming MD simulation time series into spatial heat maps. The original purpose of this approach was to detect allosteric communication patterns in proteins, such as hinge bending and amino acid contact-forming and -breaking during folding and unfolding. Although this approach worked well for this purpose (Kovacs and Wriggers, 2016), one obvious limitation that impeded wider adoption was the exclusive focus on proteins. In this work, we have applied the algorithms for the first time to lipid and solvent interaction networks. This was motivated by our interest in the mechanism of the electroporation of cell membranes. This work has also prompted us to develop a faster and more robust mutual information solver that is described in the accompanying paper. Other generalizations of our heat map approach, such as to nucleic acids and mixed protein-membrane systems, are the subject of future work.

The generalization of our numerical algorithm to aqueous solvents has revealed one limitation of our contact graph approach, namely, the quadratic scaling of the coarse-grained interaction network. This was not a problem for small proteins in the past work or for the 128 lipids in this study; however, the water molecules require at least one representative atom and cannot be coarse-grained further. (Force fields that group several water atoms together, such as the Martini force field, do exist, but the adequate modeling of intruding water fingers during electroporation requires full atomic detail). The mapping of pairwise interactions *R*_*X,a*_(*i, j*) before linear compression is the main performance bottleneck. We note that our choice of relative distance geometry *X*_*i,j*_(*t*) is rooted in the lack of a fixed reference in the liquid systems considered in this study. In the future, it would be desirable to find a reference-free but linearly scaling equivalent *X*_*i*_(*t*) that is suitable for statistical comparison with the activity function.

In the application to electroporation simulations, our heat map approach highlighted a small number of important lipids. This allowed us to efficiently search the trajectories for any mechanisms or patterns. While this is ongoing research and the causality remains unclear, the preliminary results obtained thus far suggest that pore lining lipids do not actively cause pore formation; instead, they rather passively follow the water perforation, which occurs first, as was proposed earlier by Tokman et al. (2013). This interpretation agrees with the result of our solvent heat map that showed only nonspecific interactions at the solvent-lipid interface but no sign of lipids driving the solvent at the pore location. Past efforts to identify a driving mechanism have always led to initial intruding water fingers—the phospholipids fall down their potential energy hill into the membrane interior *after* the water molecules (Vernier and Ziegler, 2007; Ziegler and Vernier, 2008; Kohler et al., 2015).

Even if the water molecules (but not lipids) play a driving role, a useful signature for pore initiation (nucleation) could exist among the lipid molecules. Perhaps promisingly, we found several lipids that were not associated with the growing pore but that indirectly contributed before (and during) pore formation through a weakening of the bilayer. Because the lipids are interchangeable and the results differ between trajectories, these “supporting events” seemingly occurred at random. However, our statistics could be limited by our choice of global activity functions, and more localized activity functions (that would reflect more specific degrees of freedom) could reveal a hidden nucleation mechanism of poration that has has eluded us thus far.

In summary, the proposed methodology provides new analyses for electroporation studies by transforming the temporal time series of simulations into spatial features. Additional future practical applications of this framework could include protein-lipid systems and studies of the effect of lipid- and water-soluble agents, in which allosteric mechanisms could be directly visualized on the embedded structures, as was the case in earlier applications to protein folding and hinge detection (Kovacs and Wriggers, 2016). All tools developed for this study will be documented and released in version 1.5 of the *TimeScapes* package that is freely available on our web site, http://timescapes.biomachina.org.

## Author contributions

The *TimeScapes* analysis algorithms were designed, implemented, and applied by WW. The molecular dynamics trajectories were generated by FC. The mathematical theory of information matching (described in more detail in the accompanying paper) was created by JK. The experimental context and interpretation of this work were supervised by PV. The paper was written by WW, FC, and PV.

## Funding

WW, FC, and JK were supported in part by the Frank Batten endowment to WW and by the National Institutes of Health grant R01GM62968. PV and FC were supported in part by the Air Force Office of Scientific Research (FA9550-15-1-0517 and FA9550-14-1-0123).

### Conflict of interest statement

The authors declare that the research was conducted in the absence of any commercial or financial relationships that could be construed as a potential conflict of interest.
